# Estimating the cost of diagnosing HIV at birth in Lesotho

**DOI:** 10.1371/journal.pone.0202420

**Published:** 2018-08-15

**Authors:** M. Tchuenche, M. M. Gill, L. Bollinger, L. Mofenson, M. Phalatse, M. Nchephe, M. Mokone, V. Tukei, A. Tiam, S. Forsythe

**Affiliations:** 1 Avenir Health, Project SOAR (Supporting Operational AIDS Research), Washington DC, United States of America; 2 Elizabeth Glaser Pediatric AIDS Foundation, Project SOAR (Supporting Operational AIDS Research), Washington DC, United States of America; 3 Ministry of Health, Maseru, Lesotho; 4 Elizabeth Glaser Pediatric AIDS Foundation, Project SOAR (Supporting Operational AIDS Research), Maseru, Lesotho; The University of Warwick, UNITED KINGDOM

## Abstract

**Background:**

Infants with HIV infection, particularly those infected *in utero*, who do not receive antiretroviral therapy (ART) have high mortality in the first year of life. Virologic diagnostic testing is recommended by the World Health Organization between ages 4 and 6 weeks after birth. However, adding very early infant diagnosis (VEID) testing at birth has been suggested to enable earlier diagnosis and rapid treatment of *in utero* infection. We assessed the costs of adding VEID to the standard 6-week testing in Lesotho where coverage of PMTCT services is nearly universal.

**Methods:**

Retrospective cost data were collected at eight health-care facilities in three districts participating in an observational prospective study that included birth testing as well as at the National Reference Laboratory in Lesotho, to investigate the cost-per-infection identified. Extrapolating to the national level, it was possible to estimate the impact of VEID on the identification of HIV-infected infants.

**Results:**

The unit cost-per-VEID test in Lesotho in 2015 was $40.50. Major cost drivers were supplies/commodities (46%) and clinical labor (22%). In 2015, 66.3% of cohort study infants born at study facilities underwent VEID; one out of 199 infants had a positive HIV DNA PCR test at birth (0.5% potential *in utero* infection), yielding a cost of $8,060 per HIV-positive infant identified. Sensitivity analysis showed costs based on Lesotho costing data ranged from $810 to $16,194 per-infected child with varying *in utero* infection rates from 5% and 0.25%, respectively. With 11,157 HIV-exposed births nationally from pregnant women on PMTCT, 66.3% VEID coverage, and 0.5% *in utero* infection, 37 infants infected with HIV could have been identified at birth in 2015 and 8 early infant deaths potentially averted with immediate ART compared with waiting for 6-week testing.

**Conclusion:**

If Lesotho costing data from this pilot study were applied to different epidemic circumstances, the cost-per-infected child identified by adding VEID birth testing to standard 6-week testing was lowest when *in utero* infection rates were high (when HIV prevalence is high and PMTCT coverage is low).

## Introduction

Lesotho has the second highest human immunodeficiency virus (HIV) prevalence in the world [1]. In 2015, HIV prevalence was estimated at 22.7% [2]. HIV prevalence among women increased from 26% in 2004 to 30% in 2014, while prevalence among men remained stable at 19% over the same period [2]. HIV services for prevention of mother-to-child transmission (PMTCT) are integrated in all antenatal facilities, with universal antiretroviral therapy (ART) recommended for all HIV-infected pregnant and breastfeeding women. In 2015, 11,744 children were born to HIV-infected mothers in Lesotho, and approximately 11,157 births (assuming no multiple births) were covered by Lesotho’s PMTCT intervention, which is integrated in all antenatal care, with 95% antenatal care coverage [3].

HIV disease progresses much more rapidly in children than in adults and early initiation of treatment in children infected with HIV, within the first few weeks of life, is critical to reduce early mortality [4,5]. In the absence of treatment, mortality is as high as 68% by age 2 years in infants with perinatal (*in utero* or intrapartum) infection, with much of this risk occurring within the first months of life [6,7]. Mortality begins to rise in the first few weeks of life, with an early peak of 20–30% within the first 8 to 12 weeks of life [7–9]. Early initiation of treatment between ages 6 and 12 weeks has been shown to dramatically reduce early infant mortality by 76% and decrease HIV progression by 75% in the Children with HIV Early Antiretroviral Therapy (CHER) trial [4]; however, many infants were ineligible for the CHER trial, which enrolled asymptomatic infants with normal CD4 count, due to advanced HIV disease prior to age 6 weeks. Current World Health Organization (WHO) recommendations are for initial early virologic infant diagnostic testing (EID) of HIV-exposed infants between ages 4 and 6 weeks or the earliest opportunity thereafter [10]. The addition of birth testing, or very early infant diagnosis (VEID) to the existing early infant diagnosis (EID) standard could allow earlier HIV diagnosis and earlier ART initiation, resulting in lower mortality related to HIV in infants [5]. However, there are many questions related to implementing this approach on a broad scale, including the cost of adding another virologic test to the diagnostic algorithm as well as the turnaround time of the results.

VEID is defined as testing at birth or within the first two weeks of life in addition to the standard infant testing at four to six weeks. In Lesotho, as part of a pilot evaluation, birth Deoxyribonucleic Acid Polymerase Chain Reaction (DNA-PCR) testing was added prospectively to the standard testing algorithm at ages 6 and 14 weeks at 13 study facilities in 3 districts that were participating in an observational prospective cohort study evaluating the effectiveness of PMTCT service delivery, the PMTCT Effectiveness among Women and Infants in Lesotho (PEA-WIL) study.

While birth testing appears an attractive intervention to reduce early HIV-related mortality, there is limited information about cost and potential impact of adding another virologic test to the existing diagnostic cascade. Data on the actual cost of conducting early infant HIV diagnostic testing are limited, and analyses looking at cost-effectiveness have solely used the cost of the assay itself as the cost of the test [5].

The primary purpose of this study was to assess the cost of VEID and the cost per HIV-positive infant identified. A secondary objective was to assess the potential impact of VEID on infant deaths in Lesotho.

## Methods

### Ethics approval and consent to participate

The costing component submitted as an amendment to the PEA-WIL Study was approved by the Lesotho Ministry of Health Research and Ethics Committee (ID79-2013) and the George Washington University Institutional Review Board (IRB # 101357) in Washington, DC. All study staff involved in data collection received human subjects’ research ethics and training on the informed consent process prior to being in contact with facility and laboratory personnel who also provided informed consent prior to being interviewed/providing data for the costing study. No client or their caregiver was interviewed since the focus of this costing study was to estimate the cost of providing VEID service and therefore, written human subject consent was not necessary.

### Data collection

Eight study sites where VEID testing was added prospectively to the standard testing algorithm were purposively selected from 13 PEA-WIL sites to reflect variations by facility type (hospital, health center) and geographical region (highlands, foothills, and lowlands). To address potential data issues prior to data collection, the costing data collection instruments were piloted at two facilities, however, data from this pilot-testing were not included in the final analysis.

Both direct (direct service personnel, consumables) and indirect (support and supervisory staff at facility and district level, capital costs, maintenance and utilities) costs were collected retrospectively for January to December 2015 at the eight surveyed facilities and from the National Reference Laboratory (NRL) based in Maseru, where facilities currently send their samples for testing. Staff interviews were conducted with finance/account officers, facility managers, and nurses involved in VEID at the facility and the laboratory staff processing and performing the VEID test. Staff salary grades were used to estimate staff salaries. Since clinical personnel were already trained on EID prior to the study period, there was no training-related cost for staff to perform VEID services. Also, costs of external technical assistance and operational research activities associated with VEID services were not included.

Information was collected on the quantity and cost of consumables, equipment, furniture, transportation (including cost of transporting samples and return of paper results), utilities (e.g., water, electricity, internet, telephone, waste management, cleaning services), and the amortized construction or equivalent rental value of each facility. The costing of utilities was proportionately allocated based on the space used for VEID relative to the space of the entire facility, while costs related to the rental or construction value of the facility were also similarly apportioned to the VEID program based on the number of patients seen. Input costs shared with other services were allocated to the VEID program based on the proportion of the number of VEID patients to the total number of patients seen at the facilities.

Finally, this study leveraged existing PEA-WIL study program data on outcomes needed to calculate the potential impact of VEID on infant deaths in Lesotho. The calculation of early infant deaths averted by adding VEID testing to standard EID testing with immediate ART initiation for infants identified as infected was based on results from a meta-analysis of 12 studies. This study which included 12,112 HIV-exposed infants, found 52% of infants who were infected peri-partum (*in utero* or intrapartum) and untreated had died by age 1 year. Some of these infants would have died prior to the return of an HIV test performed at age 6 weeks since mortality rate in exposed infants is about 21% in the first 2 months of life [11].

### Data analysis

Two Excel spreadsheets were customized to calculate two key outputs, namely: the unit cost of providing VEID in Lesotho, and the cost per early infection identified and treated. The 2015 average exchange rate applied was 12.77 Lesotho Loti (LSL) per US dollar [12]. Cost per VEID test was calculated using a bottom-up approach, where inputs were listed, their costs collected, and the contribution of these costs to the overall cost was quantified [13]. The calculated VEID unit cost included level of effort, sample transportation and infrastructure costs and was used to determine the cost per early infection identified and initiated on treatment in Lesotho. These results from the pilot study were then applied to a scenario in which VEID would potentially be available nationwide in Lesotho or in places with similar epidemiological settings. A sensitivity analysis was performed using infection rates that ranged from a low of 0.25% to a high of 5%, which was based on the expert opinion of individuals familiar with a range of studies that have calculated this figure for neighboring countries. For example, in the national birth testing program in South Africa, the *in utero* infection rate was 1.1% [14].

## Results

In 2015, 199 infants enrolled in the PEA-WIL study received a VEID test from the 8 surveyed facilities in Lesotho. Test results indicated that only 1 out of 199 (0.50%) infants who received VEID had a positive HIV DNA PCR test at birth; this infant was put on treatment soon after the HIV test result was available at the facility (age 8 weeks). The infant had a confirmatory HIV DNA PCR drawn at the 6-week visit, which subsequently returned to the clinic with a negative result; however, the family moved to South Africa (continuing treatment) prior to delivery of the negative result to the caregiver and being able to obtain a third DNA PCR and was unable to be contacted (lost-to-follow-up). Hence, this infant would be viewed as having indeterminate HIV infection status as the confirmatory positive test was not available. In the overall PEA-WIL study (conducted from 2014 through 2017), of the 431 infants with a birth test result, 4 had a positive birth HIV DNA PCR test that was confirmed by a second test, for an overall *in utero* infection rate of 0.9%.

The unit cost of VEID services (including counseling, drawing blood, packing and transporting samples, and performing the test at the NRL) was determined to be $40.50. Major cost drivers were supplies and commodities, clinical labor, amortized construction cost/rental value of the facilities, and sample transportation cost. Table 1 presents the breakdown of direct (68%) and indirect (32%) costs for VEID provision in Lesotho.

**Table 1:** VEID unit costs.

	Cost (US$)	%
**Direct cost**		
Clinical Labor	9.8	22%
Drugs, supplies, and commodities	17.6	46%
*Sub-total*	27.4	68%
**Indirect cost**		
Support Staff	1.3	3%
Construction and renovation	7.1	17%
Equipment	0.3	1%
Sample transportation	4.4	11%
*Sub-total*	13.1	32%
**Total**	**40.5**	**100%**

To assess the potential impact of national scale-up of VEID in Lesotho, the following additional assessment was performed. Out of 11,744 children born to HIV-infected mothers in 2015, approximately 11,157 births (assuming no multiple births) were covered by Lesotho’s PMTCT program [3]. At the 8 facilities conducting the PEA-WIL study included in this costing analysis, 66.3% (199/300 PEA-WIL infants born at these facilities) of infants received a VEID test. While reasons for non-testing were not collected in the costing study, for the approximately 30% of children not receiving birth testing in the overall PEA-WIL study, reasons for not testing included delivery at non-study facility or delivery at home (not all women recruited for the pilot study delivered at a study facility), or study-specific reasons (e.g. PEA-WIL study nurse not being present to perform the test).

If we assume that at a minimum, a national program might test a similar proportion of HIV-exposed infants at birth, then nationally 7,401 HIV-exposed children would receive VEID testing. Of the HIV-exposed infants in our study, 0.5% of these were found to have a positive HIV DNA PCR test at birth; in the overall PEA-WIL cohort undergoing birth testing, 0.9% of infants had confirmed *in utero* HIV infection. Thus, using a conservative estimate based on 66.3% VEID coverage nationally, and 0.5% *in utero* infection rate 37 HIV-positive infants could have been identified, linked to, and potentially initiated on immediate ART treatment, thus benefiting from this service if VEID had been offered nationally in 2015. Hence, a national VEID program with immediate initiation of ART would theoretically have averted 8 to 16 early deaths among infants that would have occurred prior to the standard six-week testing performance, assuming a 21% mortality rate.

The total incremental cost of adding a VEID test at birth in 2015 in Lesotho, assuming nationwide scale-up, was projected to have been $299,741 ($40.50 per recipient times 7,401 children tested at birth). Thus, dividing the incremental cost by the number of beneficiaries (given a 0.5% *in utero* infection rate) yields $8,060 as the cost per infected child identified and potentially benefiting from early treatment initiation through VEID. This same result is obtained by simply multiplying the VEID unit cost by the total number of infants who received the test during the pilot phase ($40.50 times 199). Our study focused on the actual costs of VEID, including staffing and infrastructure, and did not collect data on health care costs, while a cost-effectiveness analysis was beyond the scope of the study.

### Sensitivity analysis

Given that the data were obtained from a cohort study restricted to women enrolled in antenatal care as part of Lesotho’s Option B+ program, it is uncertain what the coverage would be if VEID services were scaled up nationally in the context of a general program. For this reason, a sensitivity analysis was carried out by varying the *in utero* infection rate from 0.25% to 5%. Outside of the PEA-WIL study, HIV-positive women may not access antenatal care, not receive HIV testing to know their HIV status, or not receive antiretroviral therapy, and hence a higher rate of *in utero* infection may be seen in the general population. For example, in review of the national birth testing program in South Africa, the rate of *in utero* infection was 1.1% [14], and in the overall PEA-WIL cohort, the rate of confirmed HIV infection was 0.9%. VEID coverage was also varied based on experts opinion from a pessimistic value of 50% coverage to a very ambitious target of 100% coverage.

Table 2 provides cost outcomes of various possible scenarios when VEID coverage and the *in utero* infection rates are varied. The impact of the additional test was estimated given the current *in utero* infection rate in the pilot study (Lesotho specific values from the eight facilities surveyed for the costing study are in bold face character in Table 2), and a potential range of *in utero* infection rates, to estimate costs should there be a higher *in utero* infection rate among the general population of HIV-infected women in Lesotho or in other similar epidemiological settings. To the best of our knowledge, this estimation of unit cost-per-VEID test in Lesotho is the first study of its kind which is applied to various potential epidemic circumstances in Lesotho or elsewhere to estimate the cost per infant identified as infected at birth. While national HIV prevalence does not affect the cost per infant tested (since only infants born to women living with HIV receive a birth virologic test), sensitivity analysis results indicated that *in utero* infection rates will affect the cost-per-infected infant identified. Thus, the cost-per-infant identified will be lower in settings with high HIV *in utero* infection rate than in settings with a low HIV *in utero* infection rate (for example, VEID may potentially be more cost-effective in an environment where there is a high HIV prevalence and a low coverage of PMTCT services). Thus, as PMTCT programs reach more and more women and there are fewer *in utero* infections, VEID programs are expected to cost more per child identified as infected. Conversely, in countries with low coverage of PMTCT programs and higher *in utero* infection rates, VEID may be more cost-effective.

**Table 2:** Cost outcome of VEID (assuming different epidemic circumstances). Specific parameter values from the Lesotho pilot study are: VEID coverage 66.3%, *in utero* infection rate 0.50% and the unit cost-per-VEID test of $40.50 is used to calculate the cost per infant identified as infected at birth ($8,060).

VEID coverage	*In utero* infection rate	0.25%	0.50%	1%	2%	3%	4%	5%
50%	Early deaths averted*	2	6	12	23	35	47	59
	Total number identified as infected through VEID	14	28	56	112	167	223	279
60%	Early deaths averted*	4	7	14	28	42	56	70
	Total number identified as infected through VEID	17	34	67	134	201	268	335
**66.30%**	Early deaths averted*	4	**8**	16	31	47	62	78
	Total number identified as infected through VEID	18	**37**	74	148	222	296	370
70%	Early deaths averted*	4	8	16	33	49	66	82
	Total number identified as infected through VEID	20	39	78	156	234	312	390
80%	Early deaths averted*	5	9	19	37	56	75	94
	Total number identified as infected through VEID	22	45	89	179	268	357	446
90%	Early deaths averted*	5	11	21	42	63	84	105
	Total number identified as infected through VEID	25	50	100	201	301	402	502
100%	Early deaths averted*	6	12	23	47	70	94	117
	Total number identified as infected through VEID	28	56	112	223	335	446	558
**Cost per infant identified as infected at birth from VEID**		$16,194	**$8,060**	$4,049	$2,024	$1,350	$1,012	$810

*Assuming 21% early mortality (within the first 2 months of life) in infants with *in utero* infection that would occur before the 6-week test result would return to allow initiation of ART, and that VEID with immediate ART if *in utero* infection was identified would avert this early mortality.

## Discussion

Becquet et al. [12] highlighted the urgent need for timely assessment of HIV infection in infants to allow for early treatment initiation to prevent early HIV-related mortality. Without ART, mortality is very rapid among children infected with HIV [9]. Thus, the WHO recommendation for EID testing at age 6 weeks may not be the optimal age to diagnose perinatal HIV infections as this could miss one-fifth of perinatally HIV-infected infants [15,16]. In a modeling exercise, a birth test to detect *in utero* infection followed by a test at 6 weeks improved outcomes and was cost-effective in South Africa [5]. Several studies have suggested that in the ART era while the number of infant infections have decreased, given the effectiveness of ART in reducing intrapartum infection, the proportion of residual infections occurring *in utero* may be increasing; for example, in the Mma Bana study of maternal ART during pregnancy and breastfeeding, 72% of infant infections occurred *in utero* [17].

The cost of adding VEID prospectively to the standard EID testing algorithm at 8 study facilities in 3 districts that were participating in an observational prospective cohort study evaluating the effectiveness of PMTCT service delivery in Lesotho was calculated. Francke et al., in their cost-effectiveness model on timing of EID testing used an expert opinion cost of $25 per test performed in South Africa because no previous study had conducted a detailed unit costing of EID or VEID [5]. However, using an ingredients-based approach (where all inputs were listed, their costs collected, and the contribution of these costs to the overall cost quantified [13]), this study found that the unit cost per VEID test in Lesotho was $40.50, over 30% more expensive that in what was assumed in South Africa. Data from the surveyed facilities indicated the cost per child with a positive birth test identified via VEID (excluding the costs of pediatric treatment, PMTCT, training and external technical assistance) was $8,060. One of the major factors driving the cost is the *in utero* infection rate. Sensitivity analysis shows that as the *in utero* infection rate increases, the cost per child identified decreases.

The potential impact of a national VEID scale-up was also assessed. This research extrapolated a scenario where VEID was scaled-up nationally in 2015, with an *in utero* infection rate ranging between 0.25% (the single child with a positive PCR test at birth) to 5% (the rate of confirmed *in utero* infection in the overall PEA-WIL cohort undergoing birth testing), a total of 37 to 74 infants would have been identified as infected at birth and 8 to 16 early infant deaths could have potentially been prevented by adding a test at birth, with immediate ART if an infant is identified as infected, as compared to waiting until the 6-week test. Sensitivity analysis was also performed by varying the *in utero* infection rates to determine the cost-per- child identified. Cost-per-child identified will be lower in settings with high HIV *in utero* infections rate and higher in settings with low HIV *in utero* infection rate.

In our study, only one infant was found to have a positive PCR test at birth in the eight surveyed facilities, and that infant initiated ART soon after diagnosis. This infant was subsequently viewed as having indeterminate HIV infection status, as the confirmatory test done was negative, and a third PCR to determine final infection status is lacking. Our study was designed to determine the cost of an individual VEID test and does not take into account the need for confirmatory testing; confirmatory testing following a single positive test would be required regardless of timing of detection of infant infection (birth or at 6 weeks), and while sensitivity of birth testing is lower than testing at later time points as it only detect *in utero* infection, the specificity of the test is similar regardless of timing [18]. In an evaluation of birth testing in 5,743 HIV-exposed infants born at Rahima Moosa Mother and Child Hospital in Johannesburg, South Africa between 5 June 2014 and 31 August 2016, only 104 (1.8%) had a positive DNA PCR result at birth [19]. After confirmatory testing, 83 (81%) of infants had confirmed HIV infection, amounting to an *in utero* transmission rate of 1.4%; 8 (8%) were assigned negative HIV infection status (all 8 had isolated indeterminate PCR result at birth with at least 2 subsequent negative viral tests); 5 (5%) infants remained uncertain infection status; and 6 (6%) were lost-to-follow-up. The overall positive predictive value of a positive birth PCR test was 0.91; the probability of being confirmed as infected was 99% for infants with an initial birth positive PCR result compared to 43% for infants with an initial birth indeterminate PCR test result.

The potential benefits of VEID are dependent upon turn-around time (TAT) for test results to return to the clinic, receipt of results by the clinic, and rapid initiation of ART. A pilot study in Lesotho evaluating TAT of EID and VEID testing (which underwent the same sample processing at the central laboratory based in Maseru) found that the median TAT from birth to caregiver result receipt was not significantly different between birth and 6 weeks tests, with expedited return of test results when the test was found to be PCR-positive (TAT from blood draw to receipt of result by caregiver was 38 days); the average age at antiretroviral therapy initiation in infected infants was age 43 days with birth testing compared to age 102 days with 6-week testing [20]. In an evaluation of the introduction of birth testing at one large urban hospital in South Africa where there was expedited testing for the birth specimens (median TAT 2 days), there was an *in utero* infection rate of 1.6%, and with active outreach and liaison to other clinics, 96% of 99 infected infants were initiated on ART at a median age of 8 days [21]. However, the authors noted for infants with birth PCR-negative tests, for whom there was not active tracking, only 52% of mothers returned to receive the results. The authors also noted that human resources were critical to implementing birth testing, including having counselor presence covering seven days a week including public holidays; staff to perform neonatal phlebotomy or collect dried blood spots; and staff to perform active follow-up of infected infants.

Turn-around time can be significantly improved with use of point-of-care testing. In an evaluation of the Xpert HIV-1 point of care test at the same large urban hospital in Johannesburg South Africa discussed above, point-of care testing was associated with good performance, improved rates of result return, and reduced time to ART initiation, with 100% of 30 infants with a positive birth point of care test initiating ART a median of 5 days earlier than with standard laboratory-based birth testing. [22]. The authors noted that implementation was challenging, with similar human resource issues being important to the success of the program.

A concern that has been expressed is whether testing at birth would affect return for the 6-week EID test, which is necessary to detect intrapartum/early breastfeeding transmission. In a study of high-risk infants undergoing targeted birth testing in Cape Town, South Africa, the rate of *in utero* infected was 3.8%; infants who underwent birth testing were less likely to present for later EID compared to those who did not have a birth test, and of those who presented for later EID, they presented at a significantly older age (60 vs 50 days) [23].

### Study limitations

Despite efforts to collect all available data for a comprehensive cost analysis, this study does not purport to be exhaustive and a number of limitations should be noted. First, personnel (clinical and support staff) wages and benefits for specific workers were often not available at the facility level. Therefore, it was necessary to use salary grades provided by the Lesotho Ministry of Public Service. The average salary grade was applied to the corresponding personnel category and across all eight facilities. This approach could have potentially led to over- or under-estimation of both clinical and support staff wages.

Secondly, no time-motion study was carried out to assess how staff spent their time on VEID activities. Instead, respondents were asked to provide their level of effort, thus relying on their ability to recall how their time was spent on VEID service, possibly resulting in recall bias.

Next, basic demographics of the maternal/infant characteristics of those tested at birth and those not tested were not collected as part of the costing study; in the larger PEA-WIL cohort, the most common reasons for lacking a birth test were delivery at home or at a non-study facility or lack of staff to draw the blood. Also, the cost of quality assurance of the DNA PCR testing and the cost continuous quality improvement were not collected, thus not included in the unit cost calculation. This study was primarily focused on the cost-per-infection identified and the number of infections averted as well; the cost savings per infection averted was not investigated.

Finally, despite multiple requests, some facilities did not provide their utilities costs (water and electricity), and the construction cost of the NRL was not available. Given the limited data on property prices and the rental market in Lesotho, an equivalent rental value was estimated to that of a “typical” facility using the 2012 subsidized factory rental rates from the Lesotho National Development Corporation [24].

### Conclusion

The unit cost-per-VEID test in Lesotho in 2015 was $40.50. The cost-per-infected child identified by adding VEID birth testing to standard EID testing was lowest when *in utero* infection rates was high.

In settings where PMTCT programs are not well-established and HIV *in utero* infection rates are high, universal birth testing might be considered as the cost-per-identified child would be low, and reduction in early mortality through initiation of early treatment, rather than waiting until the infected child was identified by the standard six-week test result, would be high. On the other hand, in situations where PMTCT programs are already well-established and the HIV *in utero* infection rate is low, a targeted testing program of infants at high risk of *in utero* infection (e.g., no maternal antiretroviral therapy or less than four weeks of treatment during pregnancy) might be a more appropriate way of implementing birth testing. However, a targeted program may be more complex to implement on a national scale, which could result in high-risk infants missing birth testing and hence not being identified; and countries will need to consider both cost as well as implementation feasibility in designing a national VEID program.

## Abbreviations

ART: Antiretroviral therapy
CHER: Children with HIV Early Antiretroviral Therapy
DNA-PCR: Deoxyribonucleic acid Polymerase chain reaction
EID: Early infant diagnosis
HIV: Human immunodeficiency virus
LSL: Lesotho Loti
NRL: National Research Laboratory
PEA-WIL: PMTCT Program Effectiveness among Women and Infants in Lesotho
PMTCT: Prevention from mother-to-child transmission
VEID: Very early infant diagnosis
WHO: World Health Organization
